# Male Circumcision Due to Phimosis as the Procedure That Is Not Only Relieving Clinical Symptoms of Phimosis But Also Improves the Quality of Sexual Life

**DOI:** 10.1016/j.esxm.2020.100315

**Published:** 2021-02-02

**Authors:** Mateusz Czajkowski, Katarzyna Czajkowska, Karolina Zarańska, Alicja Giemza, Jakub Kłącz, Małgorzata Sokołowska-Wojdyło, Marcin Matuszewski

**Affiliations:** 1 Department of Urology, Medical University of Gdańsk, Gdańsk, Poland; 2 Department of Dermatology, Venerology and Allergology, Medical University of Gdańsk, Gdańsk, Poland; 3 Student Research Group at the Department of Urology, Medical University of Gdańsk, Gdańsk, Poland

**Keywords:** Male Circumcision, Phimosis, Erectile Dysfunction, Sexual Activity

## Abstract

**Introduction:**

Male circumcision is recognized as the most effective method of phimosis treatment. Analyzing the literature, the information about the influence of male circumcision due to phimosis for patients' subjective symptoms such as itching, burning, penile pain, pain during intercourse, and quality of sexual life is insufficient.

**Aim:**

To investigate the effect of male circumcision due to phimosis to patients' subjective symptoms, including erectile function and satisfaction with their genitals.

**Methods:**

The single-center prospective study began in January 2018 and ended in January 2020. Sixty-nine male, adult patients, who were qualified for circumcision due to phimosis, were included in the study.

**Main Outcomes Measures:**

The study outcomes were obtained using questionnaires such as visual analog scale 0-10 for itching, burning, penile pain, and penile pain during intercourse; International Index of Erectile Function (IIEF-5) and Male Genital Self Image Scale 7 (MGSIS-7) to assess the changes in patients sexual functioning.

**Results:**

Before the circumcision of the 69 patients included in the study, 59 patients (86%) reported some subjective symptoms of phimosis. The most frequent and most severe complaint was pain during intercourse, then itching and burning of the penis. Penile pain at rest was the least frequent. After 3 months from circumcision, subjective symptoms almost completely disappeared. All of 69 patients declared to have a sexual partner. 3 months after circumcision, all patients achieved significant improvement in both obtaining and maintaining an erection based on IIEF-5 score. Their sexual intercourse was more satisfying for them.

All patients suffering from phimosis were embarrassed about their genitals before surgery. 3 months after circumcision, satisfaction with genital self-image increased significantly.

**Conclusion:**

Male circumcision due to phimosis is not only relieving the clinical symptoms of phimosis, but it also improves the quality of sexual life.

**Czajkowski M, Czajkowska K, Zarańska K, et al. Male Circumcision Due to Phimosis as the Procedure That Is Not Only Relieving Clinical Symptoms of Phimosis But Also Improves the Quality of Sexual Life. Sex Med 2021;9:100315.**

## Introduction

Male circumcision (MC) seems to be one of the oldest surgical procedures, and it has been practiced since ancient times. Formerly, it was practiced for hygienic and religious reasons. In Judaism and Islam, it is a holy commandment.^1^ Nowadays, MC can be performed for medical or non-medical reasons. The most common medical reasons are phimosis, paraphimosis, penile cancer, and not responding to local treatment inflammation of glans penis or prepuce. Non-medical indications for circumcision include religious, social, cultural, and personal reasons.^2^^,^^3^

**Figure 1:**
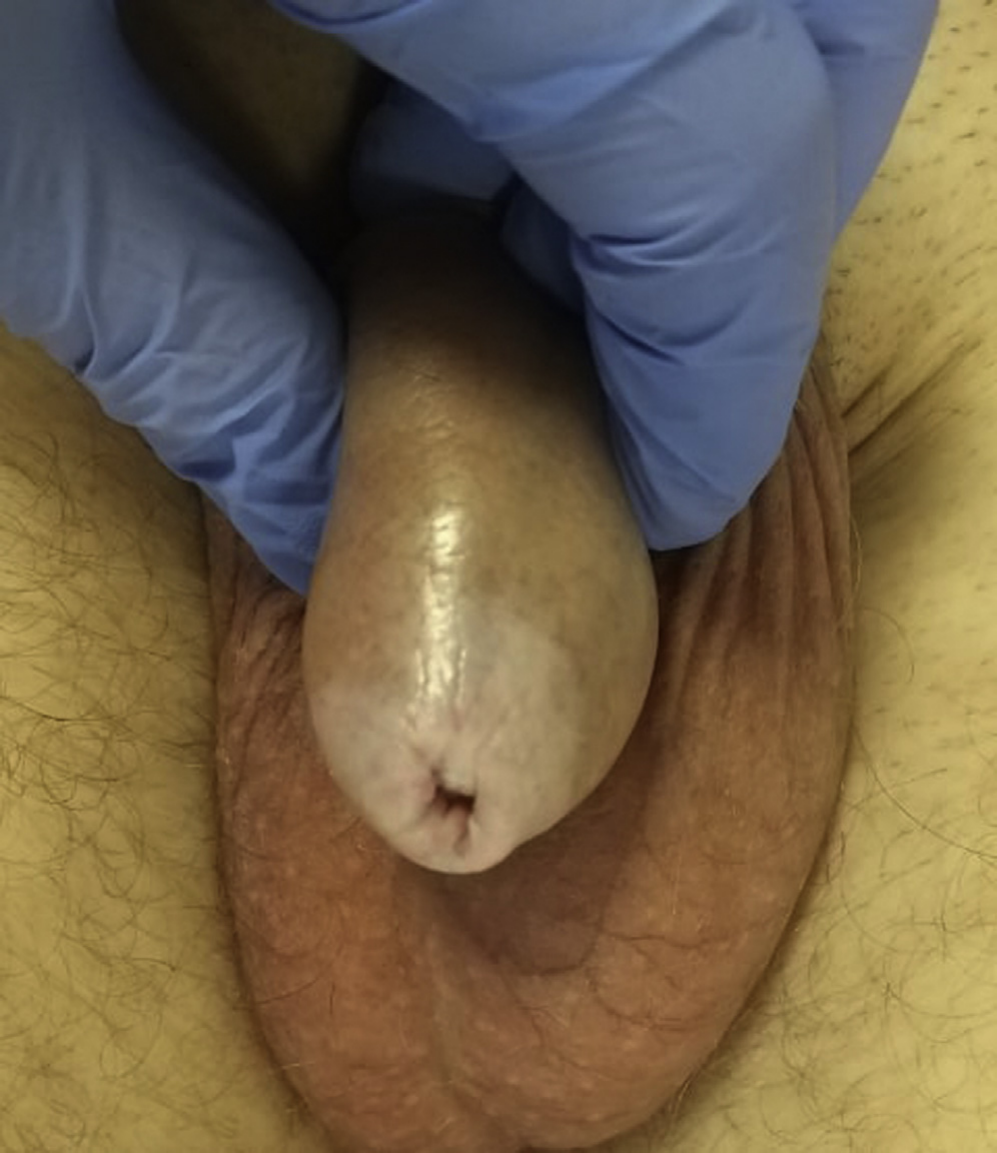
Phimosis—prepuce affected by lichen sclerosus.

Phimosis is referred to be a condition when the foreskin becomes unretractable (Figure 1). It can occur at any age and can be divided as congenital and acquired. The incidence of phimosis in the uncircumcised male population is varied, and it is estimated from 0,5 to 13%. In accordance with Morris et al, the risk of phimosis in men was 3.4% (95% CI 1.8 – 6.6).^4^

In addition, phimosis is a strong risk factor for penile cancer.^5^^,^^6^

Subjective symptoms in patients suffering from phimosis could be itching, burning, penile pain, and dyspareunia. However, despite its high incidence, in the literature, there is a lack of conclusive data about the real incidence of subjective symptoms in patients suffering from phimosis.

There are no clear guidelines on when circumcision is necessary. Noteworthy, many urologists believe that uncomplicated phimosis is not an indication for male circumcision. Moreover, patients suffering from phimosis are often afraid to undergo circumcision.

Analyzing the literature, the information about the influence of male circumcision due to phimosis for patients' subjective symptoms such as itching, burning, penile pain, pain during intercourse, and quality of sexual life is insufficient.

The main question that was attempted to answer was how male circumcision due to phimosis affects erectile function (EF), patient self-satisfaction with genitals and whether the MC resolved the subjective symptoms in a group of patients with phimosis.

## Patients and methods

The single-center prospective study was conducted from January 2018 to January 2020. An independent Ethics Committee has approved it.

69 Caucasian male, adult patients suffering from phimosis were included in the study. Informed consent for circumcision was obtained from every patient participating in the study. The patients were operated on the same urologist under local anesthesia and using sleeve circumcision technique. There were no complications such as bleeding, infection, or wound dehiscence during the study. The characteristic of the study group is presented in Table 1.

**Table 1:** Study population characteristic

Characteristic	Mean ± standard deviation	Range	*P*∗
Age (years)	45.1 ± 20	18–83	<.05
Body weight (kg)	88.8 ± 16.6	54–140	<.05
Body height (m)	1.77 ± 0.06	1.64–1.94	=.39
Body mass index (kg/m^2^)	28.4 ± 5.66	19.3–45.2	<.05

∗ Shapiro-Wilk test.

The median age of patients was 45.1 (18; 83), and the median BMI was 28.4 (19.3; 45.2). To determine clinical symptoms of phimosis, EF, and patient's satisfaction with genitals we used visual analog scale 0-10 (VAS 0-10) for itching, burning, penile pain, and penile pain during intercourse; International Index of Erectile Function (IIEF-5) and Male Genital Self Image Scale 7 (MGSIS-7) questionnaires. All patients completed questionnaires before and 3 months after the surgery.

### Statistics

The statistical analysis was performed using R version 3.4.3 statistical program.^7^ Distribution normality was determined using Shapiro-Wilk test; owing to the lack of normally distributed variables, nonparametric tests were used. Wilcoxon signed-rank test was used to compare the paired presurgery and postsurgery scores for individual patients. Subsequently, the magnitude of change from the baseline was compared between 2 and 3 groups of results using the Mann-Whitney U test and Kruskal-Wallis test, respectively. The differences were considered significant for *P*-value < .05. Data were visualized with boxplots and line plots using *ggplot2* package.^8^

## Results

All results were presented as mean and standard deviation. Before circumcision of the 69 patients included in the study, 59 patients (86%) reported any subjective symptoms of phimosis. The most frequent and most severe complaint was pain during intercourse (n = 41; 59.4%), then itching (n = 34; 49.3%) and burning (n = 33; 47.8%) of the penis. Penile pain at rest (n = 15; 21.7%) was less frequent than others. After 3 months from circumcision, subjective symptoms almost completely disappeared or were minimized (pain during intercourse n = 1, 1.4%; itching n = 4, 5.8% or burning n = 4, 5.8% of the penis; penile pain at rest n = 0). The data from the VAS 0-10 for itching, burning, penile pain, and penile pain during intercourse are presented in Table 2.

**Table 2:** VAS (0-10) results concerning itching, burning, penile pain (independent and during intercourse) before and after circumcision

Question	Before circumcision	After circumcision	*P*∗
Itching	2.6 ± 2.7	0.4 ± 1.1	<.001
Burning	2.1 ± 2.6	0.4 ± 1.4	<.001
Penile pain	0.6 ± 1.4	0.1 ± 0.9	=.015
Pain during intercourse	3.5 ± 3.2	0.2 ± 1.1	<.001

VAS = visual analog scale.

Data presented as mean ± standard deviation.

∗ Wilcoxon signed rank test.

All of 69 patients declare to have a sexual partner, but before surgery, only 45 patients (65%) found sexual satisfaction. The patients were divided into 4 groups in accordance with the severity of erectile dysfunction (ED) based on the IIEF-5 score (severe 1-7; moderate 8-11; mild to moderate 12-16; mild 17-21; no erectile dysfunction 22-25). As per this scale, before circumcision, a particular number of patients belonged to the mentioned groups: 23 (33.3%) severe; 4 (5.8%) moderate; 8 (11.6%) mild to moderate; 14 (20.3%) mild; 20 (29.0%) no ED. 3 months after circumcision, all patients achieved significant improvement in both obtaining and maintaining an erection based on IIEF-5 score. Their sexual intercourse was more satisfying for them. The declared level of satisfaction with sexual life was 87% (n = 60). The distribution of patients into groups depending to the severity of ED was as follows: 21 (30.4%) severe; 2 (2.9%) moderate; 4 (5.8%) mild to moderate; 8 (11.6%) mild; and 34 (49.3%) no ED. The results from the IIEF-5 questionnaire are presented in Table 3.

**Table 3:** IIEF-5—preoperative and postoperative results

Question	Before circumcision	After circumcision	*P*∗
1. How do you rate your confidence that you could get and keep an erection?	3 ± 1.8	3.4 ± 1.9	<.001
2. When you had erections with sexual stimulation, how often were your erections hard enough for penetration?	2.6 ± 2.1	3 ± 2.2	<.001
3. During sexual intercourse, how often were you able to maintain your erection after you had penetrated your partner?	2.6 ± 2.1	3.1 ± 2.2	<.001
4. During sexual intercourse, how difficult was it to maintain your erection to the completion of intercourse?	2.6 ± 2.1	3 ± 2.2	<.001
5. When you attempted sexual intercourse, how often was it satisfactory for you?	2.6 ± 2.2	3.1 ± 2.2	<.001
Total	13.3 ± 9.5	15.4 ± 10.2	<.001

IIEF-5 = International Index of Erectile Function.

Data presented as mean ± standard deviation.

∗ Wilcoxon signed-rank test.

All patients suffering from phimosis were embarrassed about their genitals before surgery. Most answers in the questionnaire (MGSIS-7) related to satisfaction with genitals and the comfort of showing them to others were negative. 3 months after circumcision, satisfaction with genital self-image increased significantly, and patients answered positively to the same questions from MGSIS-7. Results from MGSIS-7 questionnaire are presented in Table 4.

**Table. 4:** MGSIS-7 results before and after circumcision

Question	Before circumcision	After circumcision	*P*∗
1. I feel positive about my genitals	2.4 ± 0.8	3.3 ± 0.7	<.001
2. I am satisfied with the appearance of my genitals	2.4 ± 0.7	3.2 ± 0.7	<.001
3. I would feel comfortable letting a sexual partner look at my genitals	2.4 ± 0.8	3.1 ± 0.8	<.001
4. I am satisfied with the size of my genitals	2.7 ± 0.7	3 ± 0.6	<.001
5. I think my genitals work the way they are supposed to work	2.6 ± 0.7	3.1 ± 0.6	<.001
6. I feel comfortable letting a health care provider examine my genitals	2.3 ± 0.7	2.9 ± 0.8	<.001
7. I am not embarrassed about my genitals	2.3 ± 0.7	3.2 ± 0.7	<.001
Total	17 ± 4.3	21.9 ± 4.2	<.001

MGSIS = Male Genital Self-Image Scale.

Data presented as mean ± standard deviation.

∗ Wilcoxon signed-rank test.

## Discussion

MC is considered the most effective treatment for phimosis with efficacy estimated at nearly 100%.^9^ Generally, MC performed from all medical and non-medical indications usually does not lead to severe complications. However, if the side effects occur, bleeding and infection are the most common.^10^ Despite that, many candidates for circumcision are often afraid of this procedure. The most common fears are fear of pain, bleeding, hypersensitivity of the penis glans, diminishes of their sexual pleasure, and their female sexual partners.^11^^,^^12^ Not all urologists believe that circumcision is necessary in all cases of phimosis. This is most likely because not all patients with phimosis will have clinical symptoms. In our study, of the group of 69 patients enrolled in the research, 10 patients did not report any subjective symptoms of phimosis.

To our best knowledge, there is a lack of research using VAS to investigate the impact of male circumcision due to phimosis to alleviate typical symptoms of phimosis such as itching, burning, penile pain at rest, and during sexual intercourse. This kind of test seems to be valuable as a tool for urological consultation because some of the male patients suffering from phimosis are afraid of circumcision harmful effects. In our study, the most frequent complaints before circumcision were pain during intercourse (VAS 3.5 ± 3.2), itching (VAS 2.6 ± 2.7), and burning (VAS 2.1 ± 2.6). Only a few patients reported penile pain without any stimulation (VAS 0.6 ± 1.4). After surgery, all clinical symptoms of phimosis disappear or were minimized (Table 2).

The role of the prepuce, which is removed during circumcision in sexuality is not clear. It is an integral part of the external genitalia, and it is covering the glans of the penis. Somatosensory innervation becomes from the dorsal nerve of the penis.^13^ Some authors suggest that loss of prepuce during male circumcision leads to bad consequences such as decreased penile sensitivity due to denervation and keratinization of penile glans.^12^ According to Kim et al, who conducted research with 373 sexually active men (255 circumcised vs 118 uncircumcised), male circumcision decreases sexual enjoyment and masturbatory pleasure. Complications after male circumcision or the loss of nerve endings are likely causes of this phenomenon.^11^ Moreover, some opponents of circumcision mention many functions of the foreskin, which could be lost during circumcision. Some of them are the protection of the adult glans from damage and loss of sensitivity, storage, and release of the natural lubricant, providing sensory feedback, and preventing the painful erections and orgasm.^13^^,^^14^ By contrast, a systematic review conducted by Cox et al claimed that free nerve endings do not correlate with sexual response. Furthermore, the penile sexual sensation is higher after MC because there is better access to penile glans during sexual intercourse.^15^ Moreover, a recent systematic review conducted by Morris et al indicates that MC has no negative impact on sexual life. In addition, data supporting adverse effects of MC sexual function, sensation, and pleasure becomes from low-quality studies.^16^ Additionally, it is claimed that women prefer circumcised men as sexual partners. The main criteria they mention are better appearance, better hygiene, reduce risk of infection, greater sexual enjoyment, and enhanced sexual activity.^17^

The meta-analysis by Yang et al assessed the influence of MC on premature ejaculation, intravaginal ejaculation latency time, the difficulty of orgasm, ED, and pain during intercourse. There were no significant differences in premature ejaculation and difficulty of orgasm. However, in the group of circumcised men were significant lower ED, pain during intercourse and intravaginal ejaculation latency time.^18^ The same Tian et al in systematic review and meta-analysis investigated the effect of MC on male sexual functions. There were no significant differences in sexual desire, dyspareunia, premature ejaculation, ejaculation latency time, ED, and orgasm difficulties. However, the authors claimed these results should be evaluated in light of the low quality and heterogeneity various studies.^19^

Yang et al compared EFs in the large group of 442 young adults before and 90 days after circumcision did not achieve significant differences between the mean total IIEF-5 (IIEF-5 score: before surgery 22.56 ± 1.74 vs 22.69 ± 1.49 after surgery; *P* = .141). It is worth noting that the confidence in obtaining and maintaining an erection increased significantly in patients after circumcision.^20^ Similarly, Masood with colleagues examining a group of 84 men undergoing circumcision (IIEF-5 score: before surgery 22.41 ± 0.94 vs 21.13 ± 3.17 after surgery; *P* = .4) and Xia et al in the research with the group of 81 patients (IIEF-5 score: before surgery 21.7 ± 5.6 vs 20.9 ± 6.2 after surgery; *P* = .402). In both studies, differences between mean IIEF-5 score were not significant.^21^^,^^22^ In another study, among 4,456 sexually experienced men in a randomized trial of male circumcisions, 2,210 were immediately circumcised, and in 2,246, circumcision was delayed for 24 months. There were no observed significant differences in EFs.^23^ Opposed to the cited research Chinkoyo et al in the study conducted in Lusaka, from 478 participants divided into 2 groups circumcised and uncircumcised. Comparing these groups, circumcised patients had a significantly higher total IIEF-5 score (*P* < .005).^24^

In our prospective study, we compared EFs and evaluation of genital self-image at the patients before and 3 months after circumcision. The vast majority of patients enrolled in the study achieved significant improvement in all aspects of sexual life from EFs to the positive self-genital image.

There was a significant betterment between the total mean IIEF-5 score before and 3 months after circumcision (IIEF-5 score: before surgery 13.3 ± 9.5 vs 15.4 ± 10.2 after surgery). Moreover, patients enrolled in the study achieved improvement in all aspect of EFs presented in Table 3. Our results differ from those obtained in other studies. The main reason for differences between our results and those in cited studies seem to be the qualifying criteria for patient's enrollment.

Following Yang et al study, the range of age was 19-35 years,^22^ Masood et al: 18-60 years,^23^ Xia et al: 22-54 years^22^ The Ugandan study enrolled 4,996 patients whose range of age was 15-49 years.^25^ The same in Zambian study: the range of age was 18-59 years.^24^ The indications of circumcision in cited studies varied consist of phimosis, balanitis, human immunodeficiency virus prevention, painful erections, cosmetic reasons, the sexual partner demand, and for better personal hygiene.^20^, ^21^, ^22^, ^23^, ^24^ In our research, the patients were more diverse in terms of age (18-83 years old), and phimosis was the only indication for surgery. It could be the main reason why we observed significant improvement in all aspects of the IIEF-5 questionnaire. Moreover, comparing our study, IIEF-5 total preoperative score was significantly lower than in cited studies (IIEF-5 score: before surgery 13.3 ± 9.5). It probably depends on the fact that 59 from 69 patients included in this study reported clinical symptoms of phimosis, and it depends on terms of the age study population. Male circumcision is not only relieving of clinical symptoms of phimosis but also significantly improves all aspects of EFs (IIEF-5 score: after surgery 15.4 ± 10.2).

The good self-image of the genital area is an essential part of psychological well-being. In the group of patients with penile abnormalities, genital self-image has been decreased. To investigate the relation between the appearance of phimosis and genital perception, we used Male Genital Self-Image Scale-7 (MGSIS-7).^25^ According to the authors’ best knowledge, there is no other research concerning the relationship between male circumcision and genital self-image using MGSIS-7 questionnaire in the literature. In our study before circumcision, patients rather felt pessimistic about their genitals (24 ± 0.8), did not satisfy with the appearance of their genitals (2.4 ± 0.8), did not feel comfortable letting a sexual partner look at their genitals (2.4 ± 0.8), did not satisfy with the size of their genitals (2.7 ± 0.7), did not think that their genitals work the way they supposed to work (2.6 ± 0.7), did not feel comfortable letting a health care provider examine their genitals (2.3 ± 0.7), and they were embarrassed about their genitals (2.3 ± 0.7). 3 months after the procedure, the genital self-image improved significantly, as shown in the data presented in Table 4. The main reason for this fact could be the removal of the foreskin which was causing phimosis. The exposed glans of the penis is easier to maintain proper hygiene. Therefore, they do not feel ashamed to show the external genitalia to their partner or medical staff. Moreover, phimosis causes the situation that the prepuce traps the penis. For this reason, after surgery, patients evaluate their penile as much bigger than before.

## Conclusion

Male circumcision in patient suffering from phimosis relieved all clinical symptoms of phimosis. Moreover, it was able to improve sexual life by better EF and positive genital self-image. This knowledge, important for the patient, will certainly become beneficial for urologists during the explanation process before the patient signs the consent to the circumcision due to phimosis.

## Statement of authorship

Mateusz Czajkowski, Conceptualization, Investigation, Formal Analysis, Acquisition of Data, Writing - Original Draft, Writing - Review & Editing; Katarzyna Czajkowska, Methodology, Investigation, Project Administration, Writing - Review & Editing; Karolina Zarańska, Acquisition of Data, Writing - Review & Editing; Alicja Giemza, Acquisition of Data, Writing - Review & Editing; Jakub Kłącz, Acquisition of Data, Writing - Review & Editing; Małgorzata Sokołowska-Wojdyło, Conceptualization, Writing - Review & Editing; Marcin Matuszewski, Conceptualization, Writing - Review & Editing.
